# Conditions of Failure

**Published:** 1905-06

**Authors:** E. K. Wedelstaedt

**Affiliations:** St. Paul, Minn.


### CONDITIONS OF FAILURE.[1]

BY E. K. WEDELSTAEDT, ST. PAUL, MINN.

Mr. President and Gentlemen,—These pictures are made from casts of a condition of failure, which I have obtained for your consideration. If you will compare the conditions represented in the pictures with those you can find existing in the casts, you will observe that the artist has painted an accurate reproduction of the faulty environment which originally surrounded the mesial and distal surfaces of that lower right second bicuspid and the adjacent first molar. If you will examine the cast containing the two gold fillings, you will see to what an extent recurrence of decay has destroyed that portion of the distal surface of the second bicuspid which lies to the buccal of the gold filling. It was not possible to show the cavity of decay that existed to the buccal of the gold filling which had been placed in the mesial surface of the molar. One of the casts, however, very fully illus-

---

[1] Read before the Academy of Stomatology, Philadelphia, December, 9, 1904.

trates this, and gives us an excellent idea of the size of the cavities after the fillings had been removed. The size of the cavities, however, can be compared with the size of the gold fillings. In making this comparison you can obtain a very good idea of the differences which existed in the size of the cavities before and after the fillings had been removed.

In September, 1903, the young man from whom I obtained the impressions from which these casts were made consulted me regarding the condition of that lower right first molar. He complained of the loss of an amalgam filling which had occupied part of the disto-occlusal surface. He was to leave the city in the evening, but wished some kind of temporary operation made. The cavity was filled with cement, and in September, 1904, he returned and made an appointment to have a somewhat different operation made. The first step toward making this operation was to obtain separation, for the contact point between the molars was at the gingival margin. The Perry separator was adjusted and the teeth lifted apart, the cavity and space filled with gutta-percha, an appointment made to make the operation, and the patient dismissed for the time being. He returned, per appointment, and after preparing the cavity I decided that a gold crown would be the best thing for the tooth. If you will examine any of the casts, you will observe that the first molar contains outlines of fillings in the occlusal, buccal, lingual, and mesial surfaces. The filling in the buccal surface was of unusual size, and that portion of the crown to the occlusal of the filling did not have the appearance of being very strong. Besides this, I found a cavity of decay to the buccal of the gold filling that had been placed in the mesial surface, which made it necessary to remove that filling. In consulting with the patient, however, I found that he wished both cavities filled. The cavity in the disto-occlusal surface called for a very extensive operation, and on account of the condition of the tooth just spoken of I was unwilling to make the operation with gold. An amalgam filling was therefore made in the cavity in the disto-occlusal surface, as is illustrated in Figs. 2 and 5. A week later the amalgam filling was polished, and then an impression of the parts was taken. Thereafter several impressions were taken. The casts from the impressions are herewith presented for your examination.

I next turned my attention to the faulty conditions existing in the mesial surface of the molar and the distal surface of the adjacent bicuspid. (You will observe from the illustrations, as well as the conditions existing in the casts, that the original operator had a penchant for placing his point of contact at the gingival margin. In fact, I may as well tell you now that the same state of affairs exists among fillings in the teeth which are in three other parts of this young man's mouth. Evidently his former dentist is not making a comprehensive study of the conditions with which he deals.) The separator was adjusted and the teeth moved until there was ample space between that molar and bicuspid in which to operate and make fillings which would restore the parts to a condition of normality. While the separator was still in place the fillings were removed and the cavities, as well as the space, filled with pink base gutta-percha. (The filling in the disto-occlusal surface of the bicuspid was loose.) Prior to adjusting the separator a piece of gutta-percha was placed between the molar and bicuspid, and then an impression taken. After the impression was obtained the gutta-percha was removed, the separator adjusted, space obtained, and the fillings removed and placed in their places in the impression with the gutta-percha. The casts are just as they came from the impressions. They have not been trimmed. The cavity of decay in the distal surface of that bicuspid was not cut. It is precisely as it came from the impression. If you will examine Cast 4, you will observe there are still some remnants of the modelling compound left in the interproximal space between the bicuspid and molar. A week or ten days after space had been obtained between the molar and bicuspid the young man came, per appointment, and I at once took an impression with the gutta-percha *in situ*. On removing the impression the gutta-percha was removed and placed in the impression. This is how I obtained the casts containing the cavities. The cavity in the mesio-occlusal surface of the molar was first filled, and later on the cavity in the disto-occlusal surface of the bicuspid was filled. On completion of the operations another impression was taken. Two views have been made of each of the occlusal surfaces, for the reason that it is not possible to shade with gold paint. Besides this, I was very desirous that this particular case be very fully understood.

For a number of years I have been asking the members of our profession that they study with some care the causes which lead to the failure of dental operations. From time to time I have illustrated my remarks with pictures similar to these which are before us. I feel that some good has resulted, and at the present time a few men are thinking along these lines with perhaps more thought than they have previously given this subject. Considerable work of this kind is being done by the men living in the Northwest. Perhaps fifty or sixty men are now taking impressions, and some of these men have a collection of casts of many conditions of failure. My own collection contains about a thousand casts; it may be a trifle more or less. There must also be two hundred or more of just such pictures as I have here, which are used to illustrate the conditions I may speak of, in an effort to interest other practitioners in this work. In talking to these men I say, Observe the conditions as they exist between this second bicuspid and first molar. You all certainly know that decay in the teeth of the young begins at or near the point of contact. After decay has progressed for a while the break in the continuity of the arch causes the teeth to move more closely together. As the teeth move together the interproximal space is constantly being narrowed. If decay is allowed to progress and no attention be given it, the interproximal space is very often entirely obliterated. The teeth contact from the gingival margin to the occlusal surface. Very frequently there is not a point of contact, but a contacted surface. This is an abnormal condition, and before any permanent filling is made in those cavities the teeth should be returned to their normal positions. Notice the point of contact between the first and second molars. The contact there is just below the occlusal surface. There is neither a lingual nor a buccal embrasure between the second bicuspid and first molar. Observe the embrasures here between the first and second molars. Perhaps both the conditions are better illustrated here in Diagram 3. Why was not the disto-lingual portion of that filling in the second bicuspid properly trimmed? A careful examination of the fillings in the casts seems to prove that the man used a rotary disk in the dental engine. With this rotary disk he could not trim the filling to form and give it a proper contour, provided he ran the disk between the fillings from the occlusal surface to the gingival margin. Examine the cast of this condition. Look for a moment at the filling in the disto-occlusal surface of that second bicuspid. The gold extends just to the disto-bucco-occlusal angle. From the disto-buccoocclusal angle the filling begins to slope toward the lingual. The farther the filling extends gingivally, the greater is the slope toward the lingual. Thus, there is left on the distal surface of that tooth a very faulty condition. This condition we speak of as a condition which invites caries. I have observed, wherever this condition is made or left on any surface of a tooth, sooner or later we have a cavity of decay beside the filling. Such conditions invite disease. Food is wedged in the space between the fillings; it is held there and undergoes changes in its composition, fermentation takes place, an acid is produced, and naturally disease ensues. I have observed that decay naturally follows where such faulty conditions have been left. The man who made these operations evidently overlooked the object in filling teeth. Of this object I have spoken a great many times, and it is, or should be, to take the abnormal conditions as they come to us and return the parts to as near a condition of normality as lies in our power. The original operator in this case did not restore conditions to what is known as normal. I speak thus plainly for the reason that there is always a space at the gum line between teeth which are normal and which are normally situated. In a number of skulls which I have examined I find that the space at the gum line between the lower second bicuspid and first molar is about 1.7 millimetres. In measuring this space as I find it to exist between the same teeth in the mouths of some of my patients, I find that 1.7 millimetres closely approximates the measurement which I have obtained of this space among the teeth in skulls. I find, however, that the space which is in this plaster-of-Paris cast, between the teeth just spoken of, measures 1.8 millimetres, but I do not think that that makes any difference. You can take the condition as it exists between this second bicuspid and molar, in Diagram 3, and compare it with the condition as it exists between the same teeth in Diagram 6.[1] In Diagram 3 you have the abnormal condition pictured, and in Diagram 6 you have the normal condition pictured. At the same time you can make a comparison of the condition as it exists upon the occlusal surface between those two gold fillings, as they are pictured in Diagram 2, with the same condition as it is pictured in Diagram 6. In Diagram 2 you have no point of contact between the fillings. The lingual and buccal embrasures are too

---

[1] Diagrams not furnished for illustration.—Ed.

narrow for the purpose for which they are intended. Compare the size and shape of the lingual and buccal embrasures as they are pictured in Diagram 6 with those in Diagram 3. In Diagram 6 you have a point of contact; you have a proper contour given the fillings, consequently the interproximal space is properly contoured. In Diagram 6 we do not have any condition pictured which invites disease. In Diagram 3 you can readily see what we have. The pictures of these conditions speak for themselves. Let us turn once more to Diagram 3. The reason that we did not have any inflammation of the gum between the second bicuspid and first molar is this: The gold filling in the mesial surface of this molar was flattened linguo-buccally from the occlusal surface, gingivally to and including the gingivo-cavo-surface angle of the cavity. From the looks of both the tooth and filling, the entire mesial surface of this molar is flat. But if you will observe the condition of the gingival third of the distal surface of this second bicuspid, you will find that the operator did not pass his rotary disk to the gingival of the middle third. As a consequence, the molar moved mesially and the point of contact was practically between the gingival margins. If, however, the operator had run his disk completely through this space, so that he would have cut away the gingival margin on the distal surface of that second bicuspid, the chances are that the gum septum between the second bicuspid and molar would have been a mass of disease.

For somewhat over twenty-five years I have kept a very careful record of every dental operation which I have made. During this time a number of families have been my patients. I have, therefore, been in a position to observe what the results are where gold fillings, like these pictured in Diagram 2, have been made. Not only have I observed that recurrence of decay always takes place among similarly made operations, which I myself may have made, but it has been my observation that failure of just such operations takes place where they have been made by other men in the profession. To illustrate this a little further, permit me to say that I am a member of the G. V. Black Dental Club of St. Paul. For almost ten years the members of this Club have been carrying on a sort of continuous post-graduate work. Many of the men in this organization are also obtaining casts of the conditions of failure, and they frequently bring these casts to our meetings. A number of our men have made many casts of just such failures as I have here illustrated for your consideration. Now, the membership of the Black Club is scattered over these three Northwestern States. I am, therefore, in a position to state that the conditions of failure that I have here illustrated are not confined to any special locality, among any special set of operators, or among any special set of patients, for all our men find just one thing,—that these conditions of failure always take place where an opportunity is made or left for disease to obtain a foothold. It sometimes seems to me that altogether too many men stumble and fall regarding what constitutes an "opportunity." If we could but firmly establish our ideas regarding what *opportunity* means, and eradicate all opportunities for the recurrence of caries, there would be no necessity for any more essays on "Conditions of Failure of Dental Operations."

Where a man has a dozen casts of conditions which fully illustrate a failure of some dental operations, and he also has casts which illustrate the correction of the conditions, that man is in a position to obtain much independent knowledge, provided he studies the different conditions before him in an intelligent as well as a comprehensive manner. By taking one of the casts illustrating a failure, and obtaining an expression of opinion from four or five other men regarding the cause of that failure, he is greatly benefited by comparing the expressed ideas of others with those he may have of the causes which led to that condition. By this means he can very greatly increase his sphere of usefulness by taking advantage of the knowledge he obtains and in future avoiding the making or leaving of just such faulty conditions as may have led to the failure which he has a copy of. To the student, to the men possessed of a receptive mind, or to any person who wishes to learn, I know of nothing which so materially assists and so greatly aids in a person's advancement as having a collection of just such casts as I have presented for your consideration.

The various elements which enter into and which directly lead to failure are daily being better, as well as more fully, understood through a comprehensive study of the many conditions which lead to failure of dental operations. Among a few men this special work has been reduced to a science. These men have given up much time to a study of conditions which lead to failure. Before many years this special part of our work will be reduced to a system, and these faulty conditions will be as fully understood by all intelligent men as they are to-day by the few who are earnestly striving to do the best which can be done. Teachers will also take up this matter and give instruction regarding it, so that those leaving institutions of learning will be in a position to understand and cope with the conditions which experience has taught are pitfalls, and, therefore, to be avoided. I am of the opinion that the great advance which has been made by some of the men who are living in the Northwest can be traced directly to a continual study of such faulty conditions as are illustrated in these casts. I know of no other method which so fully brings the facts right home to men, thus making a lasting impression, as does this method of having, in plaster-of-Paris casts, copies of conditions of failure.

I thank you very sincerely for honoring me with an invitation to express my views to the members of your society. If I have interested you in this subject, then I have done all that mortal can do.

